# Paleodietary reconstruction of endemic rodents from the precolumbian Dominican Republic: Discriminating wild feeding behavior from diets linked to human niche construction activities

**DOI:** 10.1002/oa.3149

**Published:** 2022-08-07

**Authors:** Gene T. Shev, Jason E. Laffoon

**Affiliations:** ^1^ Faculty of Archaeology Leiden University Leiden The Netherlands; ^2^ Geology & Geochemistry Cluster Vrije Universiteit Amsterdam Amsterdam The Netherlands

**Keywords:** dietary mixing models, hutias, isotopic analysis, niche construction, precolumbian, zooarchaeology

## Abstract

In the Greater Antilles, certain animal taxa that have long been theorized to have been managed by indigenous peoples prior to AD1492, the main candidates being a group of endemic caviomorph rodents known as hutias (Capromyinae). This isotopic study investigates the paleodiets of several species of endemic rodents from three late precolonial sites in the northern Dominican Republic: El Flaco (cal. ad 990–1452), El Carril (cal. ad 1030–1262), and La Entrada (cal. ad 840–900) to assess whether human influence over animal diets can be determined. We examined bone collagen carbon (δ^13^C_co_) and nitrogen (δ^15^N) and tooth enamel carbon (δ^13^C_en_) isotope values of three species of hutias, *Isolobodon portoricensis* , *Isolobodon montanus* , and *Plagiodontia aedium* , alongside edible rat (*Brotomys* sp.), and domestic guinea pig ( *Cavia porcellus* ). To estimate dietary source contributions, we employed a Bayesian dietary mixing model (FRUITS v.3.0) and ran three different permutations to assess the relative contributions of C_3_ or C_4_/CAM plants. The addition of an extra 79 wild C_3_ and 40 wild C_4_/CAM plant species' isotope values from published sources to an established isotopic foodweb for the Caribbean region enabled us to discriminate between wild and domestic C_3_ and C_4_/CAM plant food sources in two of these models. Our results provide evidence of the significant consumption of domestic C_4_/CAM plants by some animals. This likely represents maize ( *Zea mays* ) consumption, which is known to have been ubiquitously cultivated by indigenous peoples in the region. This is particularly the case for *I. portoricensis* , as FRUITS modeling suggests that a few individuals consumed C_4_/CAM plants well beyond their expected natural diets as determined from feeding studies of extant hutia species. This may indicate human influence over endemic rodent diets due to niche construction activities such as horticultural practices and may reflect either opportunistic feeding on human produce or the purposeful supplementation of hutia diets by humans.

## 1 INTRODUCTION

Archaeological researchers have long suggested that indigenous peoples in the precolonial Caribbean may have been managing some animal species. These inferences are largely postulated due to the ubiquity and high frequencies of certain animals at archaeological sites, alongside indication of the translocation of these same species throughout the region by Amerindians (Allen, 1916; Flemming & MacPhee, 1999; Giovas, 2019; Giovas et al., 2016; LeFebvre, deFrance, et al., 2019; Newsom & Wing, 2004; Oswald et al., 2020; Wing, 2008, 2012). In the Greater Antillean islands of Hispaniola, Cuba, Jamaica, and Puerto Rico, certain species of the Echimyidae family (subfamily: Capromyinae) (Courcelle et al., 2019) of caviomorph rodents, known as hutias, are the primary candidates for having been managed. Hutias are a group of rodents that evolved after a single colonization event into the Greater Antilles islands during the Early Miocene, eventually diversifying into at least eight genera and 33 species over geological time to occupy a myriad of ecological niches throughout the islands (Courcelle et al., 2019; Fabre et al., 2014; MacPhee & Iturralde‐Vinent, 1995; Woods et al., 2021). In Hispaniola and Puerto Rico, one species of this radiation, *Isolobodon portoricensis*, has been speculated as undergoing “incipient domestication,” based on historical mention of corrals witnessed by European chroniclers in 16th century Hispaniola, the abundancy of hutia skeletal remains in many archaeological assemblages, and evidence of their translocation by humans between islands (de Las Casas, 1875; Newsom & Wing, 2004; Wing, 2008, 2012).

Although no physical traces of enclosures or other material evidence of captive management have been identified archaeologically, biomolecular studies have shed light on complex relations between humans and hutia. Studies have revealed evidence of zoonotic parasite transmission from hutias to humans suggesting a close cohabitation of space, (Wiscovitch‐Russo et al., 2020); provided genetic evidence of the introduction of populations of hutias to new island environs (Oswald et al., 2020; Woods et al., 2021); and highlighted similarities in dietary stable isotope values with humans which may suggest some degree of commensalism, both in the Bahamas with *Geocapromys ingrahami* and in Hispaniola with *I. portoricensis* (LeFebvre, DuChemin, et al., 2019; Shev, Laffoon, & Hofman, 2021). These studies do not necessarily indicate that hutias were held in captivity but do suggest that there was sharing of foodways and possibly indicate close cohabitation between humans and hutias.

The current research builds on previously published results of stable isotope analysis of bone collagen from archaeological *I. portoricensis* remains and other endemic taxa from four precolonial sites in the modern‐day Dominican Republic (Shev, Laffoon, & Hofman, 2021). Added to these collagen data are newly analyzed enamel isotope values, which enabled us to employ Bayesian dietary mixing modeling (FRUITS v.3.0) on several individuals. Using at our disposal three isotope proxies for each individual animal and published isotopic foodweb values of plants from Hispaniola, allows us to more succinctly ascertain whether there were distinct dietary linkages between hutias and human and sheds light on a likely mutualistic relationship.

Dietary stable isotope analysis is a useful technique for distinguishing domestic, tame, or captive diets from that of an animal's natural diet (Guiry et al., 2021; LeFebvre, deFrance, et al., 2019; Makarewicz & Tuross, 2012; Sugiyama et al., 2015, 2020). In large swathes of the Americas, agricultural maize (*Zea mays*) was a staple crop. Maize utilizes a C_4_ photosynthetic pathway resulting in plant tissues with higher carbon (δ^13^C) isotope values than all C_3_ taxa that form most the world's plant species. Therefore, in many parts of the Americas, human dietary influence over animals can be observed in unexpectedly raised δ^13^C values in animal bones and teeth, which may indicate opportunistic scavenging behaviors or the feeding of agricultural maize, or another C_4_ plant, to animals (Lee‐Thorp et al., 1989; LeFebvre, deFrance, et al., 2019; Makarewicz & Tuross, 2012; Sugiyama et al., 2015, 2020).

Human dietary influence as determined from relatively raised δ^13^C values in animal tissues may directly correlate to animal management practices (Barton et al., 2009; Monagle et al., 2018; Sharpe et al., 2018; Zavodny et al., 2015). In the Americas, human–animal interactions took on many forms that did not necessarily lead to domestication, let alone constitute a relationship where humans had direct control over animal lifeways (Zeder, 2006, 2012, 2015). Due to these often‐complicated forms of human–animal relations, we do not seek to establish from isotopic data whether hutias were being managed in captivity by indigenous peoples, let alone undergoing domestication. Human niche construction activities, such as altering plant communities by removing unwanted species and cultivating economically beneficial plants, are known to present adaptive pressures or benefits to animal species that inhabit an environment (Boivin et al., 2016; Odling‐Smee et al., 1996; Smith, 2001, 2007, 2011; Zeder, 2015). In the tropical Americas, ecological and anthropological studies indicate that some species, particularly dietary generalists or synanthropes, can benefit from and be attracted to environmental changes brought on by human agricultural activities, which in turn may be hunted in close proximity to agricultural plots to which they are attracted to, a practice known as “garden hunting” (Arce‐Peña et al., 2019; Linares, 1976; Loiselle & Blake, 1992; Ramírez‐Barajas & Calmé, 2015; Smith, 2005). Therefore, interpreting dietary linkages between humans and animals as part of a teleological process towards domestication may not necessarily be a sound approach for studying past societies from much of the Neotropics. Rather, we seek to shed light on possible dietary linkages between both species that may be the result of purposeful feeding by humans, or from animals opportunistically scavenging from human settlements and feeding on garden plots, which are both in essence mutualistic behaviors that likely lead to equifinality in dietary isotope values.

### 1.1 Dietary isotope analysis of human–animal interactions in the precolumbian Caribbean

Stable isotope analyses of carbon and nitrogen establish general information about animal diets by comparing the ratio of heavier to lighter isotope values within a consumer's tissues to the values of consumed food sources. Carbon isotope values of hard and soft tissues are dictated by the particular photosynthetic processes of plant species at the base of food webs, which vary according to environmental constraints such as temperature, aridity, and light exposure (DeNiro & Epstein, 1978; Schoeninger & DeNiro, 1984). Most plant species have a C_3_ metabolic pathway and have δ^13^C values ranging between −20 and −37‰. C_4_ plants, which are more tolerant of tropical conditions, usually have values between −7 and −17‰, while arid‐adapted crassulean acid metabolism (CAM) plants demonstrate values that mostly overlap with C_4_ species (Ehleringer, 1989; Kohn, 2010; O'Leary, 1988). Nitrogen (δ^15^N) values within bone collagen are primarily derived from consumed proteins. Nitrogen values see a stepwise increase with the higher trophic positioning of an animal, with baselines originating from the δ^15^N values of plants at the bottom of a foodweb that is derived from absorbed nitrates in substrates. The trophic position of an organism can be established by examining its δ^15^N values and can be used to differentiate the consumption of terrestrial and marine foods as marine environments generally have higher basal δ^15^N values and more trophic levels in their food webs (Keegan & DeNiro, 1988; Minagawa & Wada, 1984; Schoeninger & DeNiro, 1984).

Numerous stable isotope studies have assessed human paleodiets of precolonial and early colonial period indigenous communities in the insular Caribbean (Chinique de Armas et al., 2015, 2016; Keegan & DeNiro, 1988; Krigbaum et al., 2013; Laffoon et al., 2013, 2019, 2020; Laffoon & Vos, 2011; Pestle, 2010, 2013; Stokes, 2008). Isotopic studies of animal paleomobility have been conducted to investigate the introduction or movement of animals into different Caribbean islands (Giovas, 2016; Giovas et al., 2016, 2019; Laffoon et al., 2015, 2019; LeFebvre, deFrance, et al., 2019), while only a few isotopic studies have looked specifically at animal diets in an attempt to highlight dietary linkages between the human and animals (Laffoon et al., 2019; LeFebvre, deFrance, et al., 2019; Shev et al., 2020; Shev, Laffoon, & Hofman, 2021).

Our previous study discussed the bone collagen values of hutia and other endemic animals, establishing that there was likely some degree of commensalism between humans, dogs (*Canis familiaris*), domestic guinea pig (*Cavia porcellus*), and some hutias (*I. portoricensis*) at four Late Ceramic Age (ad 500–1500) sites in the Dominican Republic (Shev et al., 2020; Shev, Laffoon, & Hofman, 2021). Nevertheless, the ability to determine the relative portions of particularly food sources that an animal consumed is limited from the study of bone collagen carbon (∂^15^C_co_) and nitrogen (∂^15^N) values alone. This is partly because bone collagen isotope values are determined mainly by dietary protein intake, compared with enamel (and bone bioapatite) ∂^13^C values that are the result of an averaging of values from all macronutrients (fats, proteins, and carbohydrates) (Ambrose & Norr, 1993; Schwarcz, 2002). There is also the issue of disentangling equifinality in isotopic values between humans and animals when only one or two isotopic proxies are examined (Fernandes et al., 2015). This may be due to different macronutrient routing mechanisms between species or due to consumption of different types of plants that have similar values. Another phenomenon is the “canopy effect” where plants of the same species residing in tropical forest canopies have higher ∂^13^C values than ground‐dwelling plants. This can lead to organisms having different isotopic values although they ate the same species of plant or having similar isotopic values in their tissues even though they ate different food sources altogether (Blumenthal et al., 2016; Kohn, 2010; Quinn, 2019; Roberts et al., 2017).

### 1.2 FRUITS

To provide a more accurate estimation of input percentages of various dietary sources, a combination of dietary isotope values from tooth enamel apatite (∂^13^C_en_) and bone collagen (∂^13^C_co_ and ∂^15^N) provides greater fidelity in predicting dietary contributions when inputting these values into a Bayesian dietary mixing model. For our study, we have employed the software Food Reconstruction Using Isotopic Transferred Signals (FRUITS v.3.0) (Fernandes et al., 2014). Bayesian dietary mixing models analyze multiple isotopic values from a studied individual against the mean or median values and associated uncertainties of the presumed food source groups they consumed, providing probabilistic estimates of the relative portions of defined food sources that an organism likely ate (Fernandes et al., 2012, 2014; Hopkins & Ferguson, 2012).

Dietary mixing model studies have been used sparsely in precolumbian Caribbean archaeological investigations (Chinique de Armas et al., 2016; Pestle, 2010). They have been employed to assess paleodiets of indigenous peoples from Tutu, Virgin Islands, demonstrating that C_3_ plants formed the majority of the diets of all inhabitants contrary to the evidence suggested from the zooarchaeological and paleobotanical data (Pestle & Laffoon, 2018). Bayesian dietary mixing models have also been employed to assess weaning ages from the study of juvenile and adult human bone collagen values from different population groups at several sites in Cuba (Chinique de Armas et al., 2017, 2022; Chinique de Armas & Pestle, 2018). Relevant to animal diets, FRUITS was employed to estimate domestic dog (*C. familiaris*) dietary inputs from El Flaco, Dominican Republic, and Morel and Cathedrale de Basse‐Terre in Guadeloupe. These results suggest that dogs had broadly similar diets to humans, which were mainly composed of C_3_ plant foods, followed in importance by terrestrial animals, C_4_ plants, and then marine animals (Shev et al., 2020).

**FIGURE 1:**
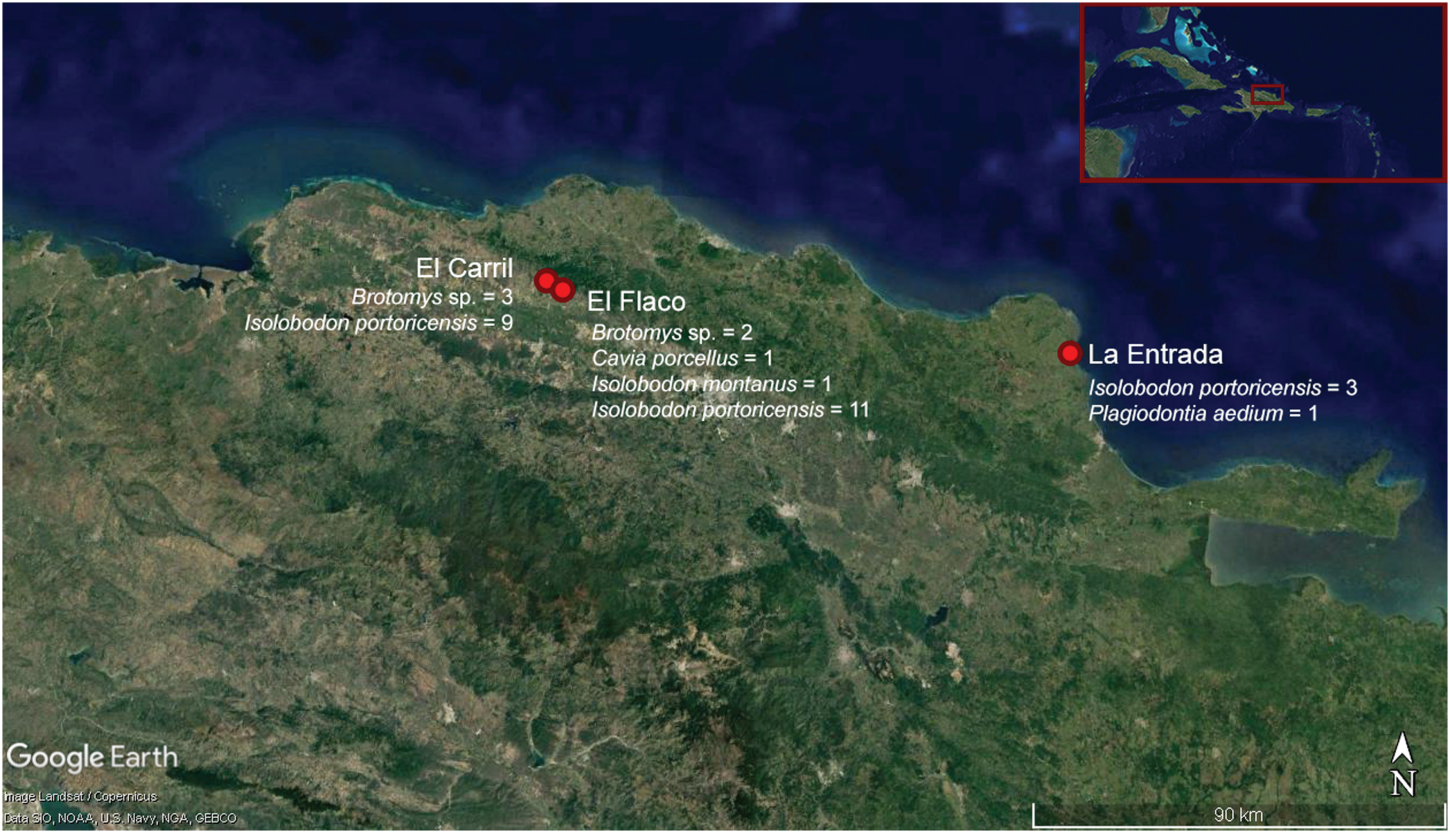
Map showing locations of the three sites in northern Hispaniola, modern‐day Dominican Republic: El Carril, El Flaco, and La Entrada. Listed are the number of individuals of each taxon from which bone collagen and tooth enamel was analyzed [Colour figure can be viewed at wileyonlinelibrary.com]

**FIGURE 2:**
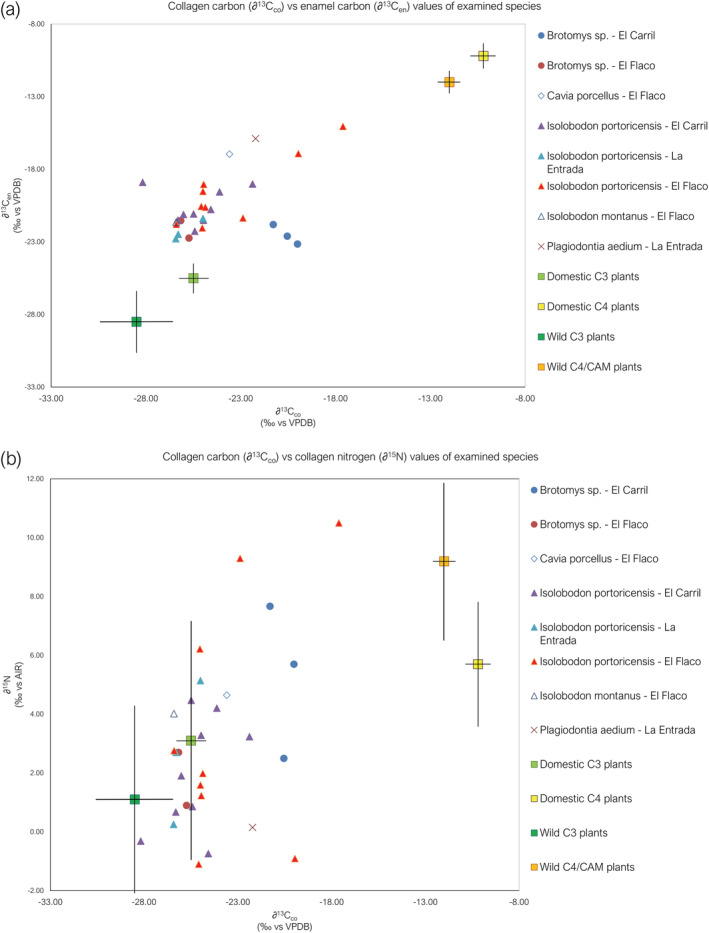
(a) Organic (collagen) and inorganic (enamel) carbon values of the studied fauna including trophic discrimination factors (TDFs). (b) Collagen carbon and nitrogen values of the studied fauna including TDFs. For both graphs, the mean isotope values are included for the four plant food source groups investigated. Bars indicate one standard deviation from the mean [Colour figure can be viewed at wileyonlinelibrary.com]

In this study, FRUITS modeling has been conducted on multiple species of endemic rodents and one domestic guinea pig (*C. porcellus*) from three precolumbian sites within Hispaniola: El Flaco, El Carril, and La Entrada (Figures 1 and 2). Examined endemic species include extinct edible rat (*Brotomys* sp.), and two species of hutia, the critically endangered Hispaniolan hutia (*Plagiodontia aedium*) and the extinct Puerto Rican hutia (*I. portoricensis*), the latter of which researchers have long speculated may have been managed by indigenous peoples. Applying Bayesian dietary mixing models to study the diets of these animals permits greater resolution in determining the probable proportions of good groups consumed; therefore, facilitating a more concise assessment of human–animal dietary entanglements than is possible from relying on one or two isotope values alone. To assess if there was a human influence on the diets of endemic animals because of niche construction activities, we made distinctions between wild and domestic C_3_ plant food sources and wild and domestic (mainly maize) C_4_ plant food sources in our FRUITS modeling.

### 1.3 The feeding preferences of hutias

To examine human–hutia commensalism, an understanding of the feeding preferences and behavioral ecology of hutias is required to ensure an accurate application of isotopic foodwebs. Particularly with “wild” animals, the isotopic values of noncultivated plant foods that they may have consumed are needed, often educated by a priori information recorded in observation studies (Bond & Diamond, 2011; Phillips et al., 2014). As *I. portoricensis*, *I. montanus*, and edible rat (*Brotomys* sp.) likely went extinct shortly after the arrival of Europeans (Turvey et al., 2007), dietary information can only be inferred from stable isotopic studies of skeletal remains and gleaned from feeding studies conducted on extant hutia species which shared morphological and functional similarities.

Cooke and Crowley (2018) studied tooth enamel isotopic values of carbon and oxygen (∂^18^O) for *I. portoricensis*, *P. aedium*, *Brotomys voratus*, and other species recovered from two Holocene cave sites on the Tiburon peninsular of southwest Haiti: Trou Jean Paul and Trouing Jérémie #5. *I. portoricensis* exhibited relatively low oxygen but intermediate carbon isotope values compared with other taxa, likely indicating a terrestrial lifestyle involving foraging in undergrowth, with isotopic values of food sources likely influenced by the “canopy effect” (Cooke & Crowley, 2018; van der Merwe & Medina, 1991). Comparatively low ∂^18^O may also indicate this species was more frugivorous than most other studied taxa. *Brotomys* sp. exhibited higher ∂^13^C values than *I. portoricensis*, but also had relatively low ∂^18^O, indicating that this species may also have been frugivorous, but more arboreal and less subject to the relatively diminished carbon values of ground‐dwelling plants due to the “canopy effect.” However, the isotopic values of these specimens may be more reflective of specific environmental constraints in southwest Haiti (Cooke & Crowley, 2018). Additionally, these two sites do not contain archaeological material, meaning that the dietary behavior of studied taxa was possibly not directly influenced by human activities, although fossil deposits at Trou Jean Paul may coincide with the Late Ceramic Age (ad 500–1500) occupation of Hispaniola by agroceramicist cultures (~1690 ± 570 cal bp) (Soto‐Centeno et al., 2017), so feasibly environmental changes brought about by humans may have affected feeding behavior of these species. At both sites, Cooke and Crowley (2018) determined that all species likely consumed some C_4_ foods, using a cutoff of ∂^13^C_en_ − 14.6‰ based on a diet‐bioapatite offset of +9.4‰ for *Rattus rattus* (Ambrose et al., 1997), accounting for the highest ∂^13^C value for C_3_ forest ecosystems of ∂^13^C −25.5‰ (Kohn, 2010) and the Suess effect (+1.5‰) (Francey et al., 1999).

Feeding studies have been conducted on seven extant species of hutia, both in captive environments and observations in the wild. Observed in natural conditions, hutias will consume 173 different plant species from 63 families. Controlled feeding studies of captive hutias, in which different plants were introduced into their diets under observation, determined that the studied hutias will readily consume 83 species of mostly cultivated forms but will also eat processed foods derived from animal meat. Most species demonstrate preferences for the consumption of tree barks, tender branches and petioles of trees and dicots, and all species were rarely observed eating monocots, especially grasses which can commonly be C_4_ plants (Gramminae) (Borroto‐Páez & Woods, 2012). All observed hutia species are primarily herbivorous but some have a tendency for omnivory, for example, Cuban hutias (*Capromys pilorides*) have been observed opportunistically catching insects and marine invertebrates in coastal habitats (Borroto‐Páez & Woods, 2012; Eisenberg & Woods, 2012; Frias & Hernandez, 1985; Manójina & Abreu, 1990). Wild *C. pilorides* favor plants from the Rosidae subclass, which includes red mangrove (*Rhizophora mangle*). Rosidae includes two families that have CAM species, Euphorbiaceae and Crussalaceae; however, no CAM species from these two families have been observed to be eaten. The majority of *C. pilorides* diets are composed of C_3_ angiosperms; they will however consume wild pineapple (*Bromelia pinguin*), a CAM plant (Borroto‐Páez & Woods, 2012; Keeley & Rundel, 2003). Species from the *Mesocapromys* genus, restricted to Cuba, also prefer plants from the Rosidae subclass, with one species *Mesocapromys melanurus* inhabiting mosaiced habitats consisting of wild and domestic plants, and favoring cultivated species within these. Jamaican hutia (*Geocapromys brownii*) has been observed eating some C_4_ grasses, such as *Panicum maximum* and *Pharus glaber*, and sugar cane (*Saccharum officinarum*) which was introduced by Europeans into Caribbean; however, these foods accounted for only 5.2% of their observed diets. The sole extant hutia from Hispaniola, *P. aedium* has been observed eating one C_4_ species, maize (*Z. mays*), although observation studies of this species in the wild are obscured by its rarity, nocturnality, and semi‐arboreal behavior (Borroto‐Páez & Woods, 2012; Eisenberg & Woods, 2012; Oliver et al., 1986). When observations have been made of *P. aedium* in the wild, they are noted to mainly consume the bark of twigs, upper branches, and trunks of wild cherry (*Prunus occidentalis*) and pine (*Pinus occidentalis*), but especially favoring the bark and fruits of wild avocado (*Persea anomala*) (Borroto‐Páez & Woods, 2012; Woods & Ottenwalder, 1992). From these studies, it is that a wide variety of plants will readily be consumed by hutias; however, there is likely very few that are C_4_/CAM species. It can therefore be deduced that for most hutia species, their wild diets are predominantly composed of C_3_ trees and other plants, which should be reflected within their dietary isotope values.

A multitude of domesticated plants were cultivated in the Caribbean region and have been identified within archaeological sites. Cultivated C_3_ species include various tree fruits, manioc (*Manihot esculenta*), sweet potato (*Ipomoea batatas*), chili (*Capsicum* sp.), zamia (*Zamia* sp.), and legumes (Fabacaea) among others, whereas evidence of C_4_ maize (*Z. mays*) consumption has been identified in starch grain and phytolith analysis, including at the site of El Flaco (Ciofalo et al., 2019, 2020; Mickleburgh & Pagan‐Jimenez, 2012; Newsom & Wing, 2004; Pagán‐Jiménez et al., 2020). Cultivated C_4_/CAM plants from the region that may have been targeted by hutia include maize, prickly pear (*Opuntia* sp.), agave (*Agave antillarum*), pineapple (*Ananas comosus*), and amaranth (*Amaranthus* sp.) (Pestle, 2010); however, the paleoethnobotanical evidence suggests that maize was like the more ubiquitously cultivated crop in precolonial Hispaniola (Ciofalo et al., 2019; Figueredo, 2015; Mickleburgh & Pagan‐Jimenez, 2012; Pagán‐Jiménez et al., 2020; Pagán‐Jiménez & Mickleburgh, 2022).

## 2 MATERIALS AND METHODS

This study involves the examination of previously analyzed collagen samples from three precolonial sites in the Dominican Republic, El Flaco (cal. ad 990–1452), El Carril (cal. ad 1030–1262), and La Entrada (cal. ad 840–900), the site histories of which are discussed in previous publications (Hofman & Hoogland, 2015; Pagán‐Jiménez et al., 2020; Shev, Laffoon, & Hofman, 2021). Accompanying these are 31 newly analyzed enamel samples from some of the same animals. Enamel from five species was analyzed; three hutia species *I. portoricensis* (*n* = 24), *Isolobodon montanus* (*n* = 1), and *P. aedium* (*n* = 1), edible rat (*Brotomys* sp.) (*n* = 5), and one example of domestic guinea pig (*C. porcellus*) that is one of only four instances of this animal recovered from precolonial contexts in Hispaniola (LeFebvre & deFrance, 2014).

Incisors were chosen over molariform teeth for enamel extraction for two reasons. Extracting adequate amounts of enamel from incisors is more easily accomplished than with molariform teeth of hutias which have thin enamel folds that disintegrate easily under a handheld rotary drill. Mandibular incisors are continually growing in all rodents, within rats at a rate of 0.4–0.6 mm per day, and within guinea pigs at 0.3 mm per day (Müller et al., 2015; Park et al., 2017). However, for many species of Capromyinae, molariform teeth are open‐rooted and continuously regenerating, which is a useful adaptation for diets that often include tough material such as tree bark (Borroto‐Páez & Woods, 2012; Hermanson & Woods, 2012). As the regeneration rates of molariform teeth in *I. portoricensis* are unknown, it was deemed that there was no extra benefit to sampling molariform teeth over incisors.

Collagen extraction protocols for previously analyzed samples are listed in that publication (see Shev, Laffoon, & Hofman, 2021). Enamel extraction follows a modified protocol from Bocherens et al. (2011). All enamel samples were taken from rodent incisors that were embedded in mandible bones that previously had collagen successfully extracted. Enamel powder was drilled from single incisors using a diamond‐tipped Dremel handheld drill and inserted into polyethylene miniature test tubes, to which 1 ml of 2% to 3% NaOCl solution was added. Samples were vortexed and sat at room temperature for 20–24 h. Bleach was removed by centrifuging samples briefly and then removing solution with a sterile pipette, 1 ml of deionized water was added to each sample which was then centrifuged, and water was subsequently removed with a pipette. This step was repeated three to four times. To remove carbonates, 1 ml of 1 M acetic acid‐CA acetate buffer (pH = 4.75) was added and samples were left at room temperature for 20–24 h. The cleaning step with deionized water was again repeated three to four times until no trace of acetic acid is left. Samples were then placed in an oven at 60°C overnight with caps open. Enamel samples were then weighed and transferred to small, labeled glass vials (minimum sample weight 0.3 mg). Samples were analyzed at the Faculty of Earth Sciences, VU Amsterdam, using a Finnigan DeltaPlus Isotope Ratio Mass Spectrometer subsequent to reaction of the samples with H_3_PO_4_ [100%] and isolation of produced CO_2_ on a Gasbench II universal automated interface. All δ^13^C, δ^18^O, and δ^15^N values are stated in δ notation in parts per thousand (‰), carbon and oxygen relative to the international Pee Dee Belemnite (PDB) standard, and nitrogen relative to the international Ambient Inhalable Reservoir (AIR) standard.

### 2.1 FRUITS protocol

FRUITS (v.3.0) was chosen due to its proficiency in integrating isotopic, elemental, and food macronutrient data, as well as uncertainties in source and consumer data, and its ability to discriminate animal‐derived foods that are rich in protein and fat from plant sources which are generally carbohydrate rich but protein poor. A comprehensive overview of the application of FRUITS for the Caribbean region is listed in Pestle and Laffoon (2018).

There are important differences in dietary behavior, macronutrient routing and trophic fractionation calculations between rodents and humans that need to be considered. Before inputting all data into FRUITS, values were altered to reflect diet–tissue offsets, also known as trophic discrimination factors (TDFs), which are the average difference between the stable isotope values of foods consumed and the tissues of the consumer. As different animals undergo distinct processes of macronutrient routing, errors can occur using Bayesian dietary mixing models when assuming TDFs. Reliable TDFs must be obtained from controlled feeding studies of species that are taxonomically or functionally analogous (Bond & Diamond, 2011; Healy et al., 2017; Kurle et al., 2014; Rio et al., 2009). As three of the five species examined in this study are extinct, generic rodent feeding studies provided our TDFs. Measured isotope values were converted to account for fractioning with a diet–tissue discrimination factor of +9.9‰ for ∂^13^C_en_ following a previous application for rodents which was based on laboratory feeding studies (Ambrose & Norr, 1993; DeNiro & Epstein, 1978; Grimes et al., 2004). For ∂^15^N_co_, a TDF of +3.6‰ ± 1.4‰ was applied based on numerous feeding studies conducted on omnivorous animals, including rodents (Ambrose, 2002; DeNiro & Epstein, 1981; Howland et al., 2003; Sponheimer et al., 2003). TDFs for ∂^13^C_co_ were calculated using the linear regression formula outlined by Pestle et al. (2015). On average, ∂^13^C_co_ TDFs were +4.9‰ ± 1.2‰ and ranged from +3.8‰ to +6.7‰. All data were input into FRUITS accounting for an instrumental uncertainty of 0.1‰.

Some limitations of using dietary mixing models need to be considered when assessing model outcomes. The inclusion of too many sources may skew results (Cheung & Szpak, 2020; Fernandes, 2016; Phillips et al., 2014; Stock et al., 2018), so we have limited our models to a maximum of four source groups composed of only plant species. There is also potential for the missing of a source group having significant effects on model outcomes (Fernandes, 2016; Stock et al., 2018); however, we rely on observational feeding studies that indicate that the Caribbean rodents we are studying are likely herbivorous, so we deemed that there was no need to include terrestrial or marine meats into our models. Another consideration is that the bioturnover rates between bone collagen and tooth enamel are vastly different. Collagen bioturnover has the slowest rate of any body tissue and likely represents an averaging of dietary sources over a long time (Gineyts et al., 2000), so when comparing collagen to tooth enamel values, particularly with rodent incisors that constantly remodel this needs to be taken into consideration. Therefore, using enamel and collagen isotope values together in a mixing model might mean an averaging of isotopic values from various time periods (Cheung & Szpak, 2020). As most species examined in this study are extinct, we have no information regarding their bone collagen bioturnover rates and tooth growth durations, so considering that we are not examining temporality or seasonal variations in diet we deem this as a minor limitation.

### 2.2 Caribbean isotopic foodweb values

Isotopic values of established food sources of plants and animals consumed by indigenous peoples in the Caribbean were garnered from previous publications (Keegan & DeNiro, 1988; Pestle, 2010; Schwarcz et al., 1985; Stokes, 2008; von Fischer & Tieszen, 1995) but were altered to only contain plants found in Hispaniola and to accommodate presumed feeding behavior of the studied taxa (Table S1). According to captive feeding studies and observations in the wild, hutia species are generally herbivorous, but with some instances of opportunistic omnivory with certain observed hutia species such as *C. pilorides*. As it is likely that only some hutias are opportunistically carnivorous, we have worked under the assumption that any consumption of animal foods would be negligible; therefore, we have omitted terrestrial and marine animal food sources from our models. As hutia and edible rats were likely not subsisting solely or predominantly on cultivated plants, published isotopic values of wild plant species with natural ranges encompassing Hispaniola were added, including an extra 79 values of C_3_ plants and 40 C_4_/CAM values. These include some epiphytes, native C_4_ amaranth species (*Amaranthus* spp.) and native grass species as possible wild C_4_/CAM sources. When possible, published values of wild species were gleaned from studies conducted on Caribbean islands; however, due to a lack of prior research on isotopic baselines for the region, values of wide‐ranging species were taken from studies in the tropical Neotropics, including Mexico, the Guianas, and Peru as long as these species had natural ranges encompassing Hispaniola (Table S1).

Three models were run: a two‐source model examining the input of C_4_/CAM and C_3_ plants; a three‐source model with C_3_ plants and dividing C_4_/CAM plants into domestic (maize and *Amaranthus amaranthus*) and wild species; and a four‐source model dividing both C_4_/CAM and C_3_ plants into wild and domestic species. The mean and standard deviations of stable isotope values of food source groups are as listed in Table 1. According to Student's *t* tests, there is a significant difference between the mean carbon values of wild and domestic C_3_ plants (*t*(79) = −6.58425, *p* = 0.00001) and between wild and domestic C_4_ plants (*t*(40) = 9.99412, *p* = 0.0001). These significant differences allowed us to treat these as separate food source groups within FRUITS (Phillips et al., 2014). The reasons for the difference between wild and domestic plant species are unknown and may be accounted for by sampling biases; however, we also theorize it might be related to the “canopy effect.” Domestically grown plants may have been cultivated in environments that were subject to land clearance and likely had less canopy cover compared with forest‐dwelling wild plants; so therefore, had higher carbon values.

**TABLE 1:** Number of samples and mean isotopic values for the six food source groupings used in this study

Food group	No. of ∂^13^C samples	Mean ∂^13^C value (‰ vs. VPDB)	No. of ∂^15^N samples	Mean ∂^15^N value (‰ vs. N_AIR_)
C_3_ plants	186	−26.8 ± 3.5	103	2.3 ± 4.4
C_4_/CAM plants	68	−11.5 ± 1.4	35	7.5 ± 4.4
Domestic C_3_	107	−25.5 ± 1.8	62	3.1 ± 2.1
Wild C_3_	79	−28.5 ± 4.3	41	1.1 ± 6.2
Domestic C_4_/CAM	28	−10.2 ± 1.5	17	5.7 ± 2.7
Wild C_4_/CAM	40	−12.0 ± 1.3	18	9.2 ± 5.2

## 3 RESULTS

### 3.1 Model outcomes

We achieved results from all three models with varying degrees of predictive accuracy and usefulness for our research aims of assessing dietary linkages between humans and animals. Listed below are the source contribution estimates generated by FRUITS, including an assessment of the utility from each of the two‐, three‐, and four‐source models (Tables 2 and 3).

**TABLE 2:** Median estimated percentages of dietary contribution sources for each sample according to the two‐, three‐, and four‐source models

	Two‐source model				Three‐source model						Four‐source model							
	C_3_	SD	C_4_/CAM	SD	C_3_	SD	C_4_/CAM domestic	SD	C_4_/CAM wild	SD	C_3_ domestic	SD	C_3_ wild	SD	C_4_/CAM domestic	SD	C_4_/CAM wild	SD
*Brotomys* sp.																		
CA8	78.25%	12.26%	21.75%	12.26%	70.30%	5.88%	11.20%	9.13%	18.51%	8.03%	31.24%	23.53%	48.26%	19.02%	8.90%	6.38%	11.60%	7.90%
CA10	69.25%	9.43%	30.75%	9.43%	64.57%	7.03%	12.88%	10.81%	22.55%	9.17%	34.49%	20.77%	43.97%	21.57%	10.84%	7.44%	10.70%	7.26%
CA24	78.73%	10.77%	21.27%	10.77%	71.58%	7.13%	14.32%	9.47%	14.11%	8.88%	53.82%	24.64%	28.45%	21.92%	9.36%	6.87%	8.36%	6.18%
FL1774	77.83%	9.34%	22.17%	9.34%	77.04%	7.93%	11.92%	8.24%	11.03%	8.07%	27.83%	22.43%	51.04%	19.45%	10.92%	7.40%	10.21%	7.63%
FL4025	74.02%	7.54%	25.98%	7.54%	72.54%	7.73%	14.77%	8.51%	12.68%	9.11%	20.31%	15.59%	53.68%	10.18%	14.08%	9.61%	11.94%	8.46%
*Cavia porcellus*																		
FL1664	61.21%	13.67%	38.79%	13.67%	56.45%	6.09%	21.24%	12.85%	22.31%	13.05%	24.54%	21.50%	37.99%	19.97%	19.41%	12.00%	18.06%	13.18%
*Isolobodon montanus*																		
FL3504	81.61%	9.10%	18.39%	9.10%	75.13%	7.45%	12.23%	8.42%	12.63%	7.82%	17.39%	13.19%	57.56%	12.75%	12.84%	8.23%	12.21%	8.36%
*Isolobodon portoricensis*																		
CA13	82.52%	9.64%	17.48%	9.64%	76.19%	6.79%	12.25%	7.58%	11.56%	8.61%	11.03%	8.07%	69.24%	10.66%	11.06%	8.37%	8.66%	6.88%
CA15	57.94%	7.37%	42.06%	7.37%	61.21%	7.11%	20.15%	11.37%	18.64%	11.44%	29.90%	19.18%	31.18%	16.39%	20.87%	10.27%	18.05%	11.55%
CA17	82.38%	7.97%	17.62%	7.97%	72.40%	6.35%	15.03%	8.84%	12.57%	8.63%	42.61%	24.72%	38.79%	21.59%	8.79%	7.32%	9.81%	7.39%
CA21	78.92%	9.95%	21.08%	9.95%	74.64%	7.76%	14.19%	8.01%	11.17%	8.33%	24.07%	14.21%	50.73%	13.48%	13.16%	8.46%	12.04%	8.81%
CA23	73.25%	9.26%	26.75%	9.26%	70.43%	6.92%	16.78%	9.71%	12.79%	9.83%	14.66%	13.08%	53.87%	12.66%	16.42%	10.69%	15.05%	9.44%
CA27	85.22%	9.49%	14.78%	9.49%	74.07%	7.77%	13.31%	9.22%	12.62%	8.13%	24.29%	19.36%	55.27%	18.23%	8.72%	7.52%	11.72%	8.61%
CA30	65.31%	8.39%	34.69%	8.39%	65.70%	9.18%	18.15%	10.47%	16.14%	11.22%	29.15%	12.74%	39.94%	11.88%	14.64%	10.81%	16.27%	10.72%
CA31	79.13%	8.13%	20.87%	8.13%	70.01%	9.52%	13.89%	11.83%	16.10%	9.37%	35.36%	21.13%	42.38%	21.07%	11.40%	8.22%	10.85%	7.09%
CA32	75.41%	10.66%	24.59%	10.66%	68.16%	7.81%	14.30%	10.95%	17.55%	9.49%	29.92%	16.81%	44.20%	12.86%	12.80%	8.44%	13.08%	8.32%
EN1	76.20%	9.23%	23.80%	9.23%	74.64%	7.96%	11.45%	8.78%	13.92%	8.53%	39.42%	24.29%	40.67%	20.58%	7.83%	6.62%	12.08%	8.56%
EN6	82.93%	8.10%	17.07%	8.10%	76.52%	7.47%	11.79%	7.80%	11.69%	8.09%	40.28%	23.87%	44.10%	19.77%	8.00%	7.33%	7.62%	5.68%
EN7	74.22%	10.28%	25.78%	10.28%	69.44%	8.08%	14.04%	9.84%	16.53%	8.63%	45.56%	21.60%	30.40%	16.36%	9.07%	7.15%	14.97%	9.07%
FL1264	80.53%	10.82%	19.47%	10.82%	73.76%	7.73%	12.99%	9.71%	13.26%	8.93%	36.39%	27.99%	41.51%	23.57%	11.15%	8.62%	10.95%	8.62%
FL1421	65.67%	9.37%	34.33%	9.37%	65.23%	9.80%	15.39%	10.65%	19.38%	10.45%	12.79%	8.17%	52.35%	10.10%	12.70%	9.62%	22.17%	13.52%
FL1543	71.31%	7.83%	28.69%	7.83%	69.06%	7.23%	13.45%	10.13%	17.49%	9.04%	23.85%	14.37%	48.81%	9.98%	12.79%	10.12%	14.55%	9.23%
FL1952	57.51%	13.65%	42.49%	13.65%	50.05%	7.07%	30.85%	14.62%	19.09%	16.12%	8.17%	5.82%	39.67%	8.61%	25.53%	15.73%	26.63%	17.91%
FL1976	74.36%	9.74%	25.64%	9.74%	68.10%	7.44%	15.85%	10.71%	16.05%	10.04%	24.37%	12.22%	53.88%	13.62%	11.08%	7.59%	10.67%	8.47%
FL2009	72.74%	10.62%	27.26%	10.62%	68.91%	7.44%	15.28%	10.52%	15.81%	9.48%	23.19%	16.83%	49.86%	13.61%	12.79%	9.69%	14.16%	9.29%
FL2413	30.51%	7.28%	69.49%	7.28%	30.38%	4.47%	20.30%	15.49%	49.32%	14.07%	17.56%	7.85%	14.41%	7.92%	14.71%	9.24%	53.32%	10.64%
FL2542	70.62%	7.93%	29.38%	7.93%	69.68%	7.12%	15.02%	9.10%	15.30%	8.80%	13.61%	9.49%	53.49%	9.03%	14.43%	9.60%	18.48%	11.80%
FL26	68.90%	9.62%	31.10%	9.62%	70.75%	8.33%	15.90%	8.50%	13.34%	9.50%	19.99%	14.28%	52.02%	10.45%	15.01%	9.53%	12.98%	8.54%
FL4075	80.57%	8.52%	19.43%	8.52%	76.01%	5.96%	10.93%	7.66%	13.06%	6.85%	31.35%	22.92%	49.99%	18.50%	10.38%	6.81%	8.29%	5.95%
FL438	61.69%	8.00%	38.31%	8.00%	61.75%	6.37%	19.26%	11.51%	18.99%	11.61%	31.44%	20.52%	35.11%	15.67%	17.60%	10.74%	15.86%	11.16%
*Plagiodontia aedium*																		
EN5	59.89%	9.61%	40.11%	9.61%	61.53%	6.82%	22.74%	11.22%	15.73%	11.30%	10.78%	8.47%	51.00%	12.45%	19.33%	11.90%	18.89%	13.51%

**TABLE 3:** Mean food group contributions for each species according to each source model

Two sources				
	C_3_	SD	C_4_/CAM	SD
Brotomys sp.	75.60%	9.90%	24.40%	9.90%
*Cavia porcellus*	61.20%	13.70%	38.80%	13.70%
*Isolobodon montanus*	81.60%	9.10%	18.40%	9.10%
*Isolobodon portoricensis*	71.60%	9.20%	28.40%	9.20%
*Plagiodontia aedium*	59.90%	9.60%	40.10%	9.60%
All species	70.00%	10.30%	30.00%	10.30%

Three sources						
	C_3_	SD	C_4_ wild	SD	C_4_ dom	SD
*Brotomys* sp.	71.20%	7.10%	15.80%	9.20%	13.00%	8.70%
*Cavia porcellus*	56.50%	6.10%	22.30%	12.90%	21.20%	13.10%
*Isolobodon montanus*	75.10%	7.50%	12.60%	8.40%	12.20%	7.80%
*Isolobodon portoricensis*	67.70%	7.50%	16.60%	10.10%	15.70%	9.80%
*Plagiodontia aedium*	61.50%	6.80%	15.70%	11.20%	22.70%	11.30%
All species	66.40%	7.00%	16.60%	10.40%	17.00%	10.10%

Four sources								
	C_3_ dom	SD	C_3_ wild	SD	C_4_ dom	SD	C_4_ wild	SD
*Brotomys* sp.	33.50%	21.40%	45.10%	18.40%	10.80%	7.50%	10.60%	7.50%
*Cavia porcellus*	24.50%	21.50%	38.00%	20.00%	19.40%	12.00%	18.10%	13.20%
*Isolobodon montanus*	17.40%	13.20%	57.60%	12.80%	12.80%	8.20%	12.20%	8.40%
*Isolobodon portoricensis*	39.40%	24.30%	40.70%	20.60%	7.80%	6.60%	12.10%	8.60%
*Plagiodontia aedium*	10.80%	8.50%	51.00%	12.50%	19.30%	11.90%	18.90%	13.50%
All species	25.10%	17.80%	46.50%	16.80%	14.00%	9.30%	14.40%	10.20%

#### 3.1.1 Two‐source model results

**FIGURE 3:**
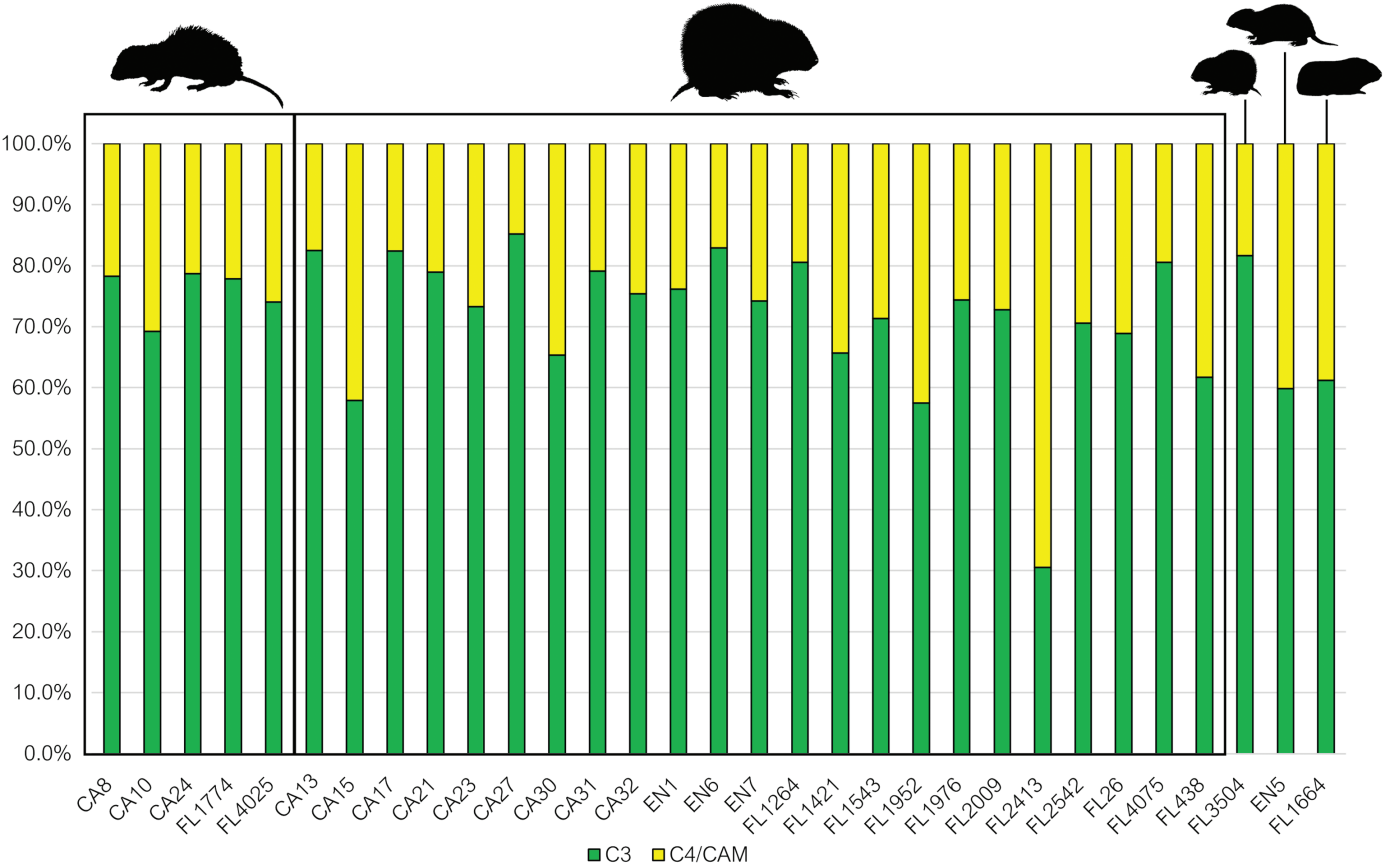
Median dietary source contribution percentage estimates according to the two‐source model. Taxa depicted from left to right are edible rat (*Brotomys* sp.), Puerto Rican hutia ( *Isolobodon portoricensis* ), montane hutia ( *Isolobodon montanus* ), Hispaniolan hutia ( *Plagiodontia aedium* ), and guinea pig ( *Cavia porcellus* ). Sample number prefixes indicate the site of origin: CA = El Carril, FL = El Flaco, EN = La Entrada [Colour figure can be viewed at wileyonlinelibrary.com]

Across all examined species, our two‐source model predicted that C_3_ plants dominated the diets of all the examined animals, except for one *I. portoricensis* from El Flaco (FL2413) of which C_4_/CAM plants comprised 69.5% of its diet (Table 3, Figure 3). The only domesticated animal in our study, guinea pig (*C. porcellus*), had a diet composed of 38.8% (SD = 13.7%) C_4_/CAM plants with only three *I. portoricensis* and the Hispaniolan hutia (*P. aedium*) from the site of La Entrada exceeding this. On average across all sites, *I. portoricensis* diets were composed of 71.6% (SD = 9.2%) C_3_ plants, which is similar to edible rat (*Brotomys* sp.) diets that on average was made up of 75.6% (SD = 9.9%) C_3_ plants. A two‐tailed Student's *t* test between these two species revealed no significant difference in their mean C_3_ plant consumption (*t*(26) = 0.72612, *p* = 0.47425), suggesting similar diets. These two species both form the majority of terrestrial vertebrate remains recovered from the site of El Flaco (Shev, Ali, et al., 2021).

Although no direct information regarding the consumption of domesticated plants are provided by the two‐source model, two noteworthy conclusions can be drawn: C_4_/CAM plants comprised more of the diets of all examined species than what was expected from observational and feeding studies that have been conducted for hutia species; and *Brotomys* sp. and *I. portoricensis* broadly had overlapping isotopic niches.

#### 3.1.2 Three‐source model results

**FIGURE 4:**
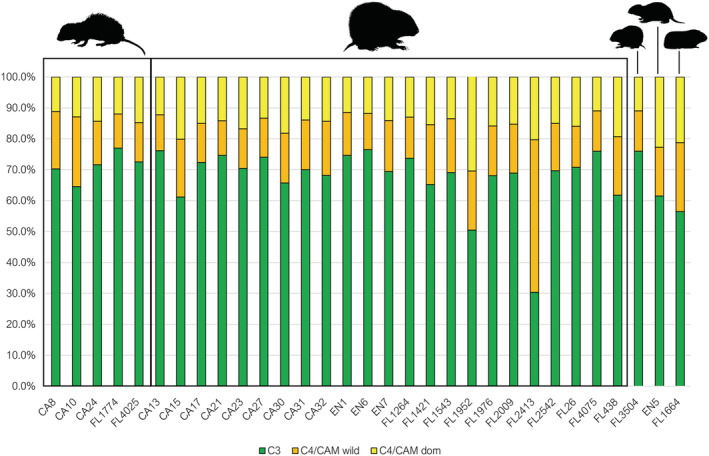
Median dietary source contribution percentage estimates according to the three‐source model. Taxa depicted from left to right are edible rat (*Brotomys* sp.), Puerto Rican hutia ( *Isolobodon portoricensis* ), montane hutia ( *Isolobodon montanus* ), Hispaniolan hutia ( *Plagiodontia aedium* ), and guinea pig ( *Cavia porcellus* ). Sample number prefixes indicate the site of origin: CA = El Carril, FL = El Flaco, EN = La Entrada [Colour figure can be viewed at wileyonlinelibrary.com]

The three‐source model provided estimates as to the proportion of C_4_/CAM wild or domestic species that comprised the diets of our examined taxa (Figure 4). On average across all sites, the two species that consumed the most domesticated C_4_/CAM plants were guinea pig, and surprisingly the endangered Hispaniolan hutia. Further analysis of more *P. aedium* samples would help us ascertain whether the frequent consumption of C_4_/CAM plants is natural for this species, as well as further study of archaeological guinea pig will aid us in establishing if this domestic species is a reliable proxy for a human‐controlled diet. The one *I. montanus* consumed the lowest amount of domestic C_4_/CAM at 12.2% (SD = 7.8%), followed by *Brotomys* sp. which averaged 13%. Three *I. portoricensis* consumed domesticated C_4_/CAM plants in amounts that exceeded 20% of their total diets (CA15 = 20.2%, SD = 11.4%; FL1952 = 30.9%, SD = 14.6%; FL2413 = 20.3%, SD = 15.5%), but for FL2413, wild C_4_/CAM rather than domestic C_4_/CAM plants formed the majority (49.32%, SD = 14.1%) of its diet.

These results suggest a varied diet for *I. portoricensis* with FL1952 and FL2413 serving as examples of two hutias with considerably high proportions of C_4_/CAM in their diets, but with different consumption rates of domestic or wild species. Importantly, 39.2% (*n* = 11) of *I. portoricensis* consumed more domestic than wild C_4_/CAM plant varieties, suggesting that for a large part of the hutia population domestic C_4_/CAM crops were a more important food source than were wild C_4_/CAM plants. The guinea pig also consumed domestic C_4_/CAM plants as 21.2% (SD = 12.9%) of their total diet but also consumed similar proportions of wild C_4_/CAM plants (22.3%, SD = 7.8%).

From the two‐source to the three‐source model across all examined species the average consumption of C_3_ plants was reduced from 70% to 66.4% of the diet. Alongside, this is a reduction in % C_3_ standard deviation from 10.3% to 7% when comparing the two‐ to three‐source models, indicating higher fidelity in dietary estimations for the three‐source model. Nevertheless, a Student's *t* test was run across all samples examining the difference in % C_3_ between the two‐ and three‐source models for *I. portoricensis*, confirming that there was no statistically significant difference (*t*(22) = 1.2114, *p* = 0.116105) in the outcomes regarding C_3_ consumption between these two models.

#### 3.1.3 Four‐source model results

**FIGURE 5:**
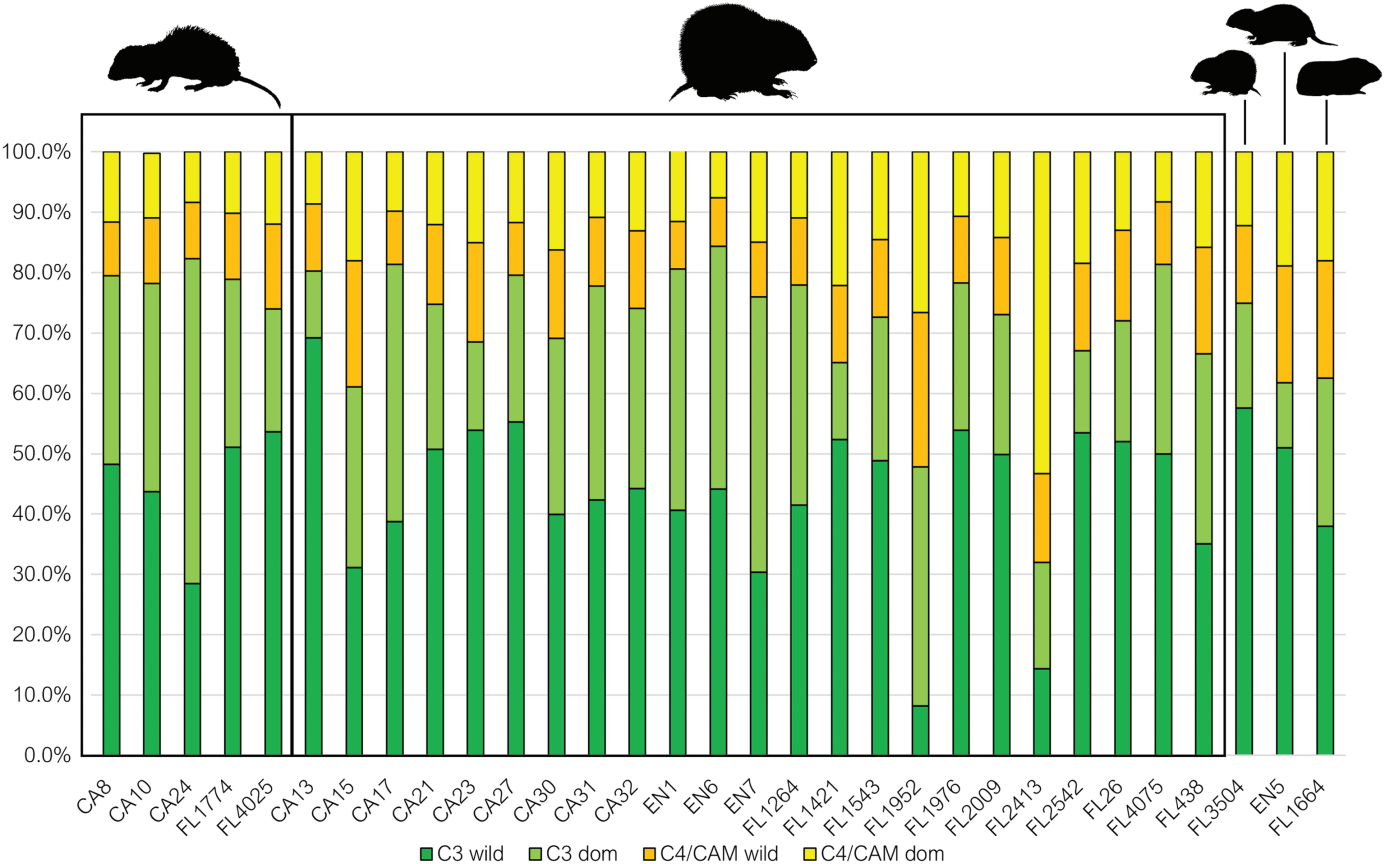
Median dietary source contribution percentage estimates according to the four‐source model. Taxa are depicted from left to right: edible rat (*Brotomys* sp.), Puerto Rican hutia ( *Isolobodon portoricensis* ), montane hutia ( *Isolobodon montanus* ), Hispaniolan hutia ( *Plagiodontia aedium* ), and guinea pig ( *Cavia porcellus* ). Sample number prefixes illustrate the site of origin: CA = El Carril, FL = El Flaco, EN = La Entrada [Colour figure can be viewed at wileyonlinelibrary.com]

In dividing C_3_ plants into domestic and wild varieties, we achieved remarkably different results compared with the two‐ and three‐source models (Figure 5). It is likely that the addition of an extra dietary source decreased the overall predictive accuracy of FRUITS (Cheung & Szpak, 2020; Fernandes, 2016; Phillips et al., 2014; Stock et al., 2018), with the standard deviations for both wild and domestic C_3_ sources being considerably high, such was the case for one *I. portoricensis* (FL1264, % domestic C_3_ = 36.4%, SD = 28%). Domestic C_3_ consumption was also high on average of all *Brotomys* sp. individuals at 33.5% of the diet, although again a standard deviation of 21.4% suggests a wide margin of error. The three individuals with the highest domestic C_3_ consumption were an edible rat (CA24, 53.8%, SD = 24.6%) and *I. portoricensis* (CA17, 42.6%, SD = 24.7%) from El Carril and *I. portoricensis* from La Entrada (EN7, 45.6%, SD = 21.6%), although the accuracy of these results again is questionable due to the high standard deviations produced in this model.

Although C_3_ wild and domestic source standard deviation ranges were high, domestic, and wild C_4_/CAM sources appeared to have performed better with smaller standard deviations, averaging SD = 9.3% for domestic C_4_/CAM and 10.2% for wild C_4_/CAM across all species. To assess differences in dietary predictions between the three‐ and four‐source models, a *t* test was run on the % domestic C_4_/CAM for *I. portoricensis* samples, confirming significant differences between the two models (*t*(23) = 2.09459, *p* = 0.020999). Counter to this, % wild C_4_/CAM contribution predictions for *I. portoricensis* were not significantly different between the three‐ and four‐source models (*t*(23) = 0.4172, *p* = 0.339279).

### 3.2 Species and site comparison

To assess if the three models provided differences according to site, *I. portoricensis* from El Flaco, El Carril, and La Entrada, and *Brotomys* sp. from El Flaco and El Carril were compared (Table 4). According to the two‐source model for both species, there was considerable overlap in mean % C_3_ and % C_4_/CAM values between El Flaco and El Carril; however, there was less consumption of C_4_/CAM by *I. portoricensis* recovered from the earlier occupied site of La Entrada (22.2%, SD = 3.7). The three‐source model predicted that hutias consumed more domestic C_4_/CAM plants at El Flaco (16.8%, SD = 5.1) and El Carril (15.3%, SD = 2.4) compared with edible rat from the same sites (13.4% and 12.8%, respectively). The four‐source model again showed that hutias ate more C_4_/CAM plants than edible rat generally.

**TABLE 4:** Comparison of *Isolobodon portoricensis* and *Brotomys* sp. mean source consumption percentages across El Flaco, El Carril and La Entrada

	Two sources				Three sources						Four sources							
	C_3_	SD	C_4_	SD	C_3_	SD	C_4_ dom	SD	C_4_ wild	SD	C_3_ dom	SD	C_3_ wild	SD	C_4_ dom	SD	C_4_ wild	SD
*Isolobodon portoricensis*																		
El Flaco	66.76%	13.28%	33.24%	13.28%	63.97%	12.49%	16.84%	5.09%	19.19%	9.79%	22.06%	8.34%	44.65%	11.24%	14.38%	4.05%	18.91%	12.00%
El Carril	75.56%	8.39%	24.44%	8.39%	70.31%	4.48%	15.34%	2.39%	14.35%	2.61%	26.78%	9.18%	47.29%	10.65%	13.09%	3.63%	12.84%	2.91%
La Entrada	77.78%	3.73%	22.22%	3.73%	73.53%	2.99%	12.43%	1.15%	14.05%	1.98%	41.75%	2.71%	38.39%	5.82%	8.30%	0.55%	11.56%	3.02%
Total	73.37%	8.47%	26.63%	8.47%	69.27%	6.65%	14.87%	2.88%	15.86%	4.79%	30.20%	6.74%	43.44%	9.24%	11.92%	2.74%	14.44%	5.98%
*Brotomys* sp.																		
El Flaco	75.93%	1.91%	24.08%	1.91%	74.79%	2.25%	13.35%	1.42%	11.86%	0.83%	24.07%	3.76%	52.36%	1.32%	12.50%	1.58%	11.08%	0.87%
El Carril	75.41%	4.36%	24.59%	4.36%	68.82%	3.05%	12.80%	1.27%	18.39%	3.45%	39.85%	9.97%	40.23%	8.51%	9.70%	0.83%	10.22%	1.36%
Total	75.67%	3.13%	24.33%	3.13%	71.80%	2.65%	13.07%	1.35%	15.12%	2.14%	31.96%	6.86%	46.29%	4.91%	11.10%	1.20%	10.65%	1.11%

The results from the three‐ and four‐source models generally indicate that *I. portoricensis* consumed more of both domestic and wild C_4_/CAM sources at El Flaco and El Carril than did edible rat. There also appears to be a difference in diet for *I. portoricensis* recovered from earlier dating La Entrada, with this species consuming less C_4_/CAM plants there than the later‐occupied, inland sites.

### 3.3 Comparisons the dietary source contributions of domestic guinea pig and wild hutia

**FIGURE 6:**
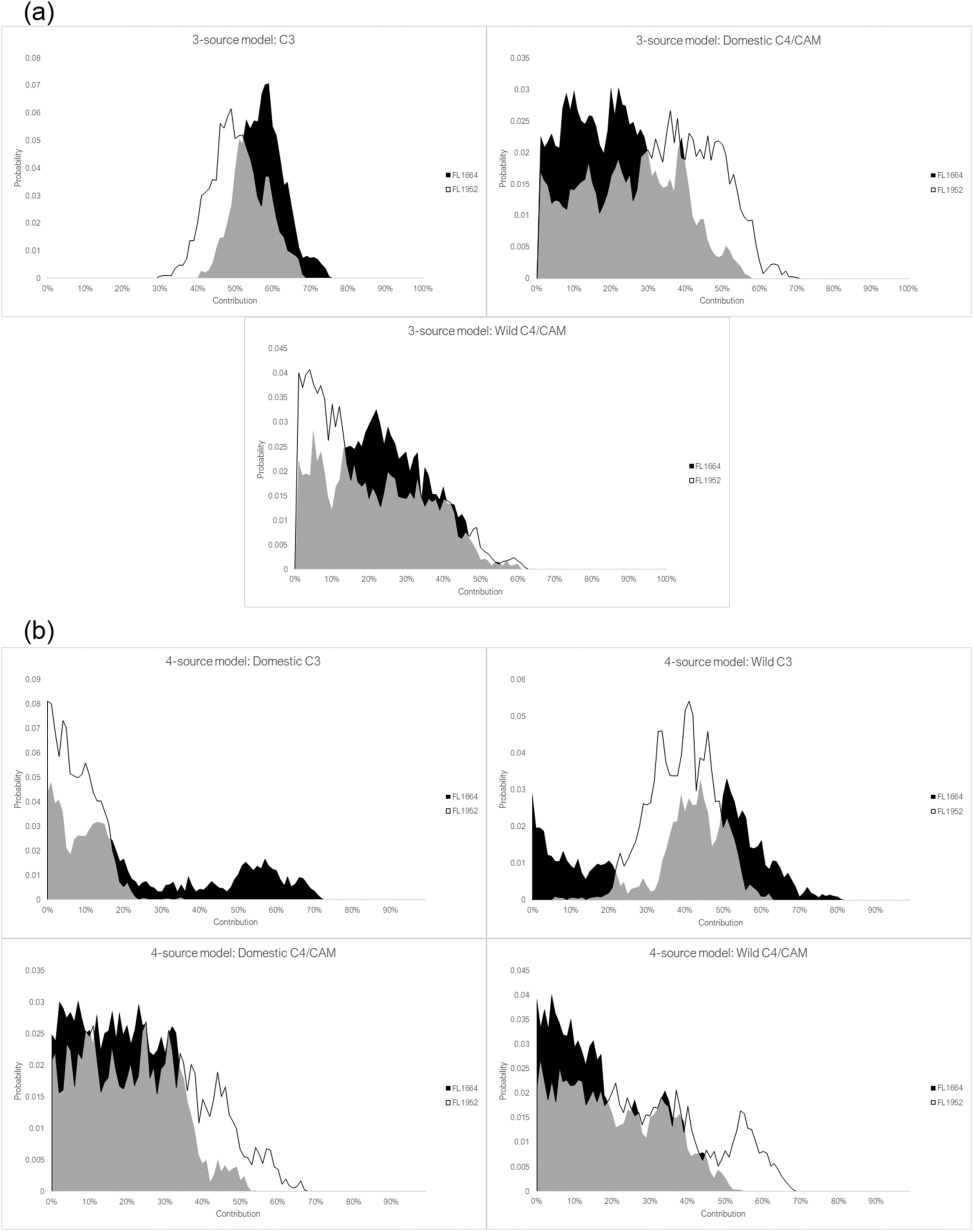
Contributions for *Isolobodon portoricensis* (FL1952) and guinea pig (FL1664) according to the (a) three‐source model and (b) four‐source model

We directly compared the dietary contribution estimates for one hutia (FL1952) that had the highest amount of C_4_/CAM consumption across all three models, and the one guinea pig recovered from El Flaco (FL1664). This was done to assess whether this one hutia had similar dietary contributions to the only domesticated species sampled in our study (Figure 6).

The three‐source model predicted overlapping contributions of all plant types between the two species, although the guinea pig likely consumed more C_3_ plants and lesser quantities of domestic C_4_/CAM plants than the *I. portoricensis*. According to the four‐source model, the hutia consumed more domestic than wild C_4_/CAM plants but compared with the guinea pig this hutia consumed greater quantities of wild C_3_ than domestic C_3_ plants. There is considerable overlap between both individuals suggesting similar diets. Nevertheless, this hutia likely consumed more domestic C_4_/CAM plants than the guinea pig according to both models, but less domestic C_3_ plants than the guinea pig according to the four‐source model; however, we consider the results for the four‐source model as less reliable than the three‐source model, which we further elaborate upon below.

## 4 DISCUSSION

All three models demonstrate some convergence regarding the premise that hutias and edible rats were consuming C_4_/CAM plants, and for the three‐ and four‐source models, that much of this was likely domesticated maize. This suggests that FRUITS is providing some accurate overview of the dietary behavior of the animals investigated, although the exact precision of the dietary estimates of each model is difficult to assess due to the extinct status of much of the fauna, and the use of independent dietary evidence gleaned from other sources. Discrepancies between models raise questions as to which model was the most accurate. The two‐source model was theoretically the most precise due to it assessing only two dietary sources, although as our aim was to assess whether animals were consuming domestic crops this model shows limited utility for our study. In actuality, the three‐source model may in fact be more accurate in determining C_3_ percentages according to the observably lower standard deviations generated from this model when compared with the two‐source model. In our opinion, the three‐source model best achieved our goal of differentiating between domestic C_4_/CAM plants (mostly agricultural maize) and wild C_4_/CAM plants. Even so, the four‐source model in our opinion is the most unreliable. Beyond the fact that increasing the amount of sources will likely decrease the predictive accuracy of FRUITS (Cheung & Szpak, 2020; Fernandes et al., 2014; Pestle & Laffoon, 2018; Stock et al., 2018), there are other reasons why the splitting of C_3_ plants into wild and domestic varieties is problematic and led to wide‐ranging predictions. First, the source plant data suggest a wider range of carbon values for C_3_ plants (δ^13^C −36.6‰ to −22.6‰, SD = 3.5) than for C_4_ plants (−16.2‰ to −9.2‰ SD = 1.4), likely reducing predictive accuracy, as when splitting C_3_ plants into two different source groups there was still high variability and overlap in isotopic values between these new groups. Second, dividing C_3_ plants into domestic and wild species ignores the possibility that some domestic varieties that have been cultivated by humans may self‐propagate in the wild without human involvement, complicating interpretations when trying to determine human influence over hutia dietary behavior. In effect, the consumption of wild or uncultivated but domesticated fruit tree species would lead to an equifinality in dietary isotope values. As domestic maize is not self‐propagating, and is an introduced species that does not occur naturally in the wild, this problem does not arise in the three‐source model that only splits C_4_/CAM plants into domestic and wild groupings.

Our study demonstrates the potential for using FRUITS or other isotopic dietary mixing models to differentiate between domestic and wild plant dietary source contributions for synanthropic herbivores. There are however some limitations we encountered which cannot be overlooked. We had to gather the isotopic values of wild taxa from a variety of sources to bolster the collection of isotopic values from published sources that constituted our isotopic foodweb for Hispaniola. Higher accuracy would be achieved if observational feeding studies of studied species can be relied upon to precisely indicate what plants an animal will consume in the wild. As three of the five studied rodent species are now extinct, this information is unfortunately unavailable; therefore, we had to rely on feedings studies of other, extant hutia species to indicate their dietary preferences. Additionally, our application of FRUITS in trying to disseminate between “wild” and “domestic” food sources is perhaps best applied for herbivores only, as the inclusion of other sources such as terrestrial or marine proteins may generate too much overlap in values and confuse dietary contribution predictions.

According to all three models, there are notable differences in *I. portoricensis* diets from the sites of El Carril and El Flaco, and that of La Entrada, with hutias from the latter site likely consuming less C_4_/CAM plants. Although this might relate to the smaller sample size of La Entrada hutias, this may also by symptomatic of differences in food availability or the intensity of agricultural production between two different regions. La Entrada is situated along the northern coastline of Hispaniola and dates to the 9th century ad, with a faunal assemblage largely composed of marine foods such as fish, shellfish, and sea turtles (Chelonidae). El Flaco and El Carril are located within viewshed of one another and are nestled in the southern foothills of the Cordillera Septentrional overlooking the agriculturally productive Cibao Valley. The inhabitants of El Flaco and El Carril likely had stronger reliance on terrestrial animals according to zooarchaeological studies (Shev, Ali, et al., 2021) and were situated within an agriculturally productive region overlooking the fertile Cibao Valley from which sediment cores provide ample evidence for anthropogenic fire usage (Castilla‐Beltrán et al., 2018, 2020; Hooghiemstra et al., 2018). It is possible that horticultural practices attracted hutias to the site and may indicate that this species was synanthropic with their predation by humans mostly reflecting garden hunting practices. This may explain the higher consumption of domestic C_4_/CAM plant in samples from these two sites. The inhabitants of La Entrada, because of its earlier date and coastal situation, may have not been as focused on agricultural production as they were on harvesting marine resources, which may be reflected in the lower percentage of C_4_/CAM plant consumption in *I. portoricensis* seen there.

The one analyzed Hispaniolan hutia (*P. aedium*) from La Entrada had the fourth highest contribution from C_4_/CAM plants. This same individual also likely ate mostly wild C_3_ plants according to the four‐source model, with equitable contributions of both wild and domestic C_4_/CAM plant sources. The high C_4_/CAM contribution is surprising, given that this species has not been observed consuming any C_4_/CAM plants apart from maize in captivity (Borroto‐Páez & Woods, 2012; Eisenberg & Woods, 2012), and had the lowest mean δ^13^C_en_ results for any hutia in the study by Cooke and Crowley (2018). We posit that the results for *P. aedium* from La Entrada may relate to an opportunistic consumption of marine foods, such as fish or shellfish, given the site's coastal location, and highlight the limitation of not including animal meats within the model. However, we acknowledge that this specimen is likely an outlier and that hutia diets are almost exclusively herbivorous. Further studies of the behavior and isotope ecology of *P. aedium* are needed to precisely assess its dietary behavior.

When we compare our findings from previously conducted feeding studies on hutias, and the isotopic niche predictions of Hispaniolan rodents by Cooke and Crowley (2018), there is overall a greater predicted consumption of C_4_/CAM sources than expected for all examined endemic species. Nevertheless, this may be an artifact of FRUITS, as the inclusion of any source will mean that it will automatically generate some percentage estimation as part of the whole diet, even if that source was never actually consumed (Fernandes et al., 2014). Cooke and Crowley's (2018) prediction of edible rat (*Brotomys* sp.) being more frugivorous may be correct as we see high estimates of C_3_ plant consumption with this species. Our three‐source model, deemed by us to be the most reliable, indicates that *I. portoricensis* on average consumed more C_4_/CAM plants than edible rat, although with a high degree of variance between individual hutias of this species. These two species appear to have some considerable overlap in isotopic niches and were likely sympatric but may have not necessarily competed for food sources, with *I. portoricensis* perhaps being more ground‐dwelling and *Brotomys* sp. more arboreal in nature (Cooke & Crowley, 2018) but with both species consuming plants with similar isotopic values. It is possible that both species were somewhat synanthropic and profited from human landscape changes such as slash‐and‐burn farming and the cultivation of garden plots and fruits trees.

The guinea pig evidently consumed significant quantities of both domestic and wild C_4_/CAM plants and can arguably be used as a proxy for what a domesticated rodent diet from precolonial contexts should look like. The FRUITS model outcomes suggest that there were no systematic feeding strategies affecting hutia diets; therefore, it is unlikely that the diets of all hutias were regimented by humans in the same way that the guinea pig likely was. Some individual hutias nevertheless were consuming C_4_/CAM plants beyond what would be expected of them from hutia feeding studies and in similar degrees to that of the guinea pig.

The *I. portoricensis* individuals that consumed high amounts of C_4_/CAM may have been consuming maize frequently enough to affect their isotopic values. It is a possibility that this may reflect some degree of dietary seasonality, with FL1952 perhaps exhibiting isotopic values that are reflective of its life cycle coinciding with maize growing and harvest seasons whereas other individuals did not. There are perhaps other scenarios in which an equifinality in isotopic values may have arisen due to unexpected dietary inputs for these extinct species. For example, it may be the case that some animals were consuming more wild plants of a variety that yields higher carbon values than the wild C_4_/CAM plants investigated in this study, contrary to what modern feeding studies would suggest about extant hutia dietary behavior. Notwithstanding these possibilities, we believe it is a more likely scenario that some individual hutias were being purposefully fed agricultural maize while most others were opportunistically feeding on maize crops. It is likely that horticultural practices such as slash‐and‐burn farming may have led to the creation of mosaic plant communities. These anthropogenic environments likely supported and bolstered populations of hutias, and possibly edible rats, which profited from these anthropogenic environments and drew them into closer contact with humans.

An expansion of this study to include samples from other sites in Hispaniola, both coastally and inland situated, would be greatly beneficial for assessing whether there were differences in food production strategies at these different locales. We expect that there may be a broad trend of more domestic plant consumption by commensal species in locations where the food production was largely focused on horticulture, compared with coastal sites that may have been more reliant on the gathering of marine resources. This method allows greater insight into the dietary effects on animals resulting from human niche construction activities beyond what can only inferred from the study of animals remains from archaeological sites at which hutia remains often comprise a significant amount of the faunal assemblage (Shev, Ali, et al., 2021).

The implementation of FRUITS to examine dietary linkages between humans and animals can be extended to other archaeological contexts and islands in the Caribbean. In the Lesser Antilles, there is some evidence to suggest that rice rats (*Oryzomys* spp.) may have also been introduced either intentionally or unintentionally to new island environments and likely served as an important food source in some islands that were relatively depauperate of other terrestrial fauna (Brace et al., 2015; Durocher et al., 2021). It is probable that these species served similar roles as hutias did in the Greater Antilles.

## 5 CONCLUSIONS

The functioning of animal management strategies at the examined sites remains opaque, and there are a multitude of explanations for why some hutias had diets that were composed of considerable amounts of domestic C_4_/CAM plants. It is probable that most hutias were attracted to and opportunistically feeding from human garden plots, given that domestic plant consumption is well demonstrated in the FRUITS modeling, and more so for *I. portoricensis* than for edible rats. This in no way indicates that all hutias were being kept in captivity, although some individuals consumed domestic C_4_/CAM plants in such high proportions that are unlikely to be the result of an unfettered opportunistic consumption of maize from garden plots; therefore, indicating that these individuals had diets that were supplemented by humans. This may constitute a degree of animal management in the form of the purposeful feeding of some hutia to possibly attract others of the same species close to human settlements.

This study highlights the applicability of multiple stable isotope analyses and dietary mixing models (FRUITS) in the study of dietary linkages between humans and endemic animals in the Neotropics. This novel approach likely has more pertinency in situations where the examined species are herbivorous, allowing us to reduce to amount of potential food source groups; therefore, increasing the predictive accuracy of the dietary mixing models. Additionally, the prevalence of C_4_ maize in the Americas as a ubiquitous food crop enabled us to easily distinguish between “wild” and “domestic” food groups in archaeological settings. Therefore, this application of FRUITS on herbivorous rodent species, like hutias and edible rats, appears to accurately convey that there was some degree of human involvement variably affecting the diets of animals at the studied sites.

## CONFLICT OF INTEREST

The authors have no conflict of interest to declare especially regarding the use of funding, the acquisition and analysis of materials the results discussed in this paper.

## Supporting information


**Table S1.** Hispaniolan isotopic foodweb values

## Data Availability

The data that support the findings of this study are available in the Supporting Information of this article.
